# Destructive and Constructive Interparental Conflict, Parenting Stress, Unsupportive Parenting, and Children’s Insecurity: Examining Short-Term Longitudinal Dyadic Spillover and Crossover Process

**DOI:** 10.3390/bs14121212

**Published:** 2024-12-17

**Authors:** Uiju Lee, Young-Eun Lee

**Affiliations:** Department of Early Childhood Education, College of Social Science, Gachon University, Seongnam-si 13120, Republic of Korea; navy512@gachon.ac.kr

**Keywords:** destructive conflict, constructive conflict, parenting stress, unsupportive parenting, child insecurity, spillover, crossover

## Abstract

Based on emotional security, stress, and spillover and crossover theories, this study aimed to examine the indirect pathways between destructive and constructive interparental conflict, parenting stress, unsupportive parenting, and child insecurity six months later. Using data from two time points beginning when Korean children (*N* = 159) were approximately 3–5 years old, two dual-mediation models of the relevant variables were constructed. The results indicate that destructive conflict is associated with higher levels of parenting stress, whereas constructive conflict is associated with lower levels of stress. Furthermore, mothers’ and fathers’ parenting stress influenced their own unsupportive parenting behaviors, which, in turn, influenced their children’s insecurity, suggesting a spillover effect. However, the crossover effect and mediation analyses provided partial support for various pathways of the hypotheses. By examining both destructive and constructive conflict, including both maternal and paternal variables, and examining not only spillover but also crossover effects, this study highlights that while constructive conflict may reduce parental stress and unsupportive parenting behaviors, the negative effects of destructive conflict may affect children more strongly. Particularly, by examining the spillover and crossover effects in the unique cultural context of Korean families, this study provides important insights into interparental conflict’s impact on child development.

## 1. Introduction

It is well established that conflict between parents is an inevitable aspect of family life. The impact of this conflict on family dynamics and child development has been extensively documented [1]. While all families experience some level of interparental conflict, the nature of this conflict and the manner in which it is resolved can have a significant impact on parenting stress and parenting styles and, in turn, on the emotional well-being of children. Destructive conflict, characterized by verbal or physical aggression, hostility, and stonewalling, is consistently associated with increased parenting stress. This can exacerbate parenting problems and lead to negative outcomes in children, including psychological insecurity and other psychological symptoms [2,3]. Conversely, constructive parental conflict, defined as behaviors such as collaboration to solve problems, has been shown to mitigate the negative effects of problematic parenting styles and foster healthier parenting attitudes [2,4].

Prior research has extensively examined the adverse effects of destructive interparental conflict on children’s development, with particular emphasis on internalizing and externalizing symptoms [1]. Moreover, the role of poor parental function as a mediator in the relationship between interparental conflict and child adjustment is well established in the literature [5,6]. Nevertheless, further research is required to elucidate the complex pathways through which constructive and destructive conflicts influence parenting stress and negative parenting behaviors, which, in turn, affect children’s long-term insecurity. In particular, there is a paucity of research that simultaneously examines the impact of these conflicts on mothers’ and fathers’ parenting stress, and subsequently on their parenting behaviors, and considers the potential spillover and crossover effects between parental stress and behavior.

### 1.1. Spillover Effects of the Mother-Father Relationship on the Parent–Child Relationship

Family systems are profoundly intertwined, and the spillover effect hypothesis [7] postulates that disruptions in one family subsystem, such as the relationship between parents, can have a cascading impact on other subsystems, particularly the parent–child relationship. The emotional and psychological toll of interparental conflict can result in increased parenting stress and unsupportive reactions, which can subsequently affect interactions with children [1,3,4]. Destructive conflict, which encompasses verbal or physical aggression, hostility, and lashing out, has been demonstrated to elevate parenting stress, resulting in more problematic parenting behaviors [3,6,8]. By contrast, constructive conflict, defined as conflict characterized by problem-solving and mutual support, has been asserted to reduce parenting stress and create a more supportive environment for children.

To gain insight into the spillover effects of dyadic conflict on children’s insecurity, we focused on mothers and fathers. Although it is probable that conflict between partners affects parenting stress and the behaviors of both mothers and fathers, which, in turn, affect their children’s insecurity, the dearth of research on fathers limits our understanding of these potential spillover effects. Specifically, based on Abidin’s [9] stress model, we hypothesized that dysfunctional parenting behaviors, such as unsupportive parental responses, mediate the relationship between parenting stress and negative child outcomes. Accordingly, we investigated the relationship between mothers’ and fathers’ interparental conflict, parenting stress, unsupportive parenting behaviors, and, ultimately, children’s insecurity.

### 1.2. Crossover Effects of Mothers and Fathers in the Relationship Between Parenting Stress and Parenting Behavior

Examination of the impact of parenting stress on parenting behavior must consider both maternal and paternal spillover effects, as well as crossover effects. The spillover effects of maternal parenting stress on parenting behavior have been extensively documented in the literature [10,11]. However, despite the fact that crossover effects, in which stress and behavior are transferred from one parent to another, can provide a more complex understanding of the interrelationships between parental stress and responses within the family system [5], scientific knowledge of these potential crossover effects is limited by the lack of studies involving both mothers and fathers [12]. Notably, even a cross-lagged model of parenting stress and child adjustment that was used to examine the longitudinal effects of Abidin’s model focused exclusively on mothers [13].

The paternal parenting vulnerability hypothesis [14] posits that fathers may be more susceptible to stress than mothers. However, previous empirical studies have yielded inconsistent results regarding paternal parenting vulnerability hypotheses [15,16,17]. As family dynamics continue to evolve, with mothers and fathers assuming increasingly shared responsibility for child-rearing [10,18], it is crucial to investigate whether they follow analogous pathways in the relationship between parenting stress and child behavior. Empirical research indicates that while both parents may engage in similar parenting behaviors, the impact of interparental conflict on stress levels and subsequent parenting can differ significantly [19,20]. Accordingly, an exploratory investigation was conducted to ascertain the distinctions and interrelationships between mothers and fathers in the pathway from parenting stress to unsupportive parenting behaviors.

### 1.3. Role of Constructive Conflict in Reducing Child Insecurity

While it is important to separately examine mothers’ and fathers’ parenting stress and unsupportive reactions, it is equally important to understand how different types of interparental conflict affect these outcomes. Prior research has concentrated on destructive conflict; however, it is not merely the existence of conflict that influences child development but rather the manner in which parents engage in conflict [21]. Constructive conflict, which is characterized by cooperation, problem-solving, support, physical affection, and a focus on resolution, has been linked to beneficial outcomes, particularly in the context of its influence on other family subsystems [8]. In contrast to destructive conflict, constructive conflict is associated with more positive child outcomes, including lower levels of externalizing and internalizing behaviors [2,21].

Although the positive influence of constructive conflict on parenting styles has been substantiated [2,8], few studies have investigated its impact on specific outcomes, such as child insecurity, particularly in the context of interparental conflict in non-Western contexts. The existing literature indicates that constructive conflict has the potential to mitigate child insecurity by mitigating the adverse effects commonly associated with destructive conflict [4,22]. However, most of these studies have been conducted in Western populations, such as those in the United States. Consequently, our understanding of how these dynamics play out in other cultural settings remains limited.

The present study examined these processes in a Korean sample, with a particular focus on the impact of constructive interparental conflict on children’s insecurity levels after six months. Considering the significant cultural differences in parenting styles and family dynamics between Western and Eastern cultures, the present study seeks to expand our current understanding of constructive conflict and its impact on child outcomes. By examining this phenomenon in the Korean context, we aim to address a crucial gap in the existing literature by offering novel insights into the potential protective role of constructive interparental conflict in the development of children’s insecurity.

### 1.4. Current Research

Although previous research has examined the relationship between interparental conflict and problematic parenting, much of this research has focused on broad age groups or adolescent populations [23,24]. Examining the relationship between interparental conflict and parental emotional socialization, particularly unsupportive reactions to children’s negative emotions, offers critical insights for the existing body of literature. Parental emotional socialization is shaped more by parents’ psychological and emotional states than by general parenting practices addressing children’s behaviors and needs [4]. Research has shown that interparental conflict tends to intensify during early childhood, a developmental stage when children assert independence and may exhibit behavioral challenges. During this period, parents’ non-supportive emotional socialization practices are likely to increase [25,26]. Moreover, parents experiencing elevated stress or emotional dysregulation due to interparental conflict may struggle to be emotionally available to their children. This lack of availability could manifest in reduced sensitivity and responsiveness, particularly toward children’s negative emotions [27]. Parents who engage in aggressive conflict resolution, silent treatment, or avoidance strategies in their interactions with partners may replicate these patterns in their parenting. For instance, they may react aggressively to their children’s negative emotions or dismiss these emotions altogether, perpetuating cycles of emotional disengagement or conflict [4,28].

Previous studies often fail to distinguish between mothers’ and fathers’ perspectives. The extant literature on parenting styles [29,30] tends to focus on specific styles, whereas other studies have concentrated on destructive conflict [6,31]. Although destructive conflict has been linked to elevated parenting stress and unsupportive responses, there has been a paucity of research examining the potential associations between constructive conflict and reduced parenting stress, and, consequently, more positive parenting. Moreover, extant studies examining constructive conflict have frequently considered mothers’ and fathers’ parenting attitudes but have not considered the mediating effects of parenting stress [8] or child outcomes, particularly child insecurity in the context of interparental conflict [2]. Building on this gap, the emotional security theory provides a valuable framework for understanding how repeated exposure to destructive conflict—characterized by uncooperative attitudes, hostile interactions, and neglect—can create a toxic environment that undermines a child’s sense of security. Particularly, empirical studies have consistently shown that among children’s various responses to interparental conflict, negative emotional responses—specifically overt emotional reactivity and behavioral dysregulation—as well as hostile internal representations, are associated with adverse effects on child development. However, the impact of children’s regulatory behaviors, such as efforts to protect or comfort their parents, on child development has shown inconsistent or contradictory results across prior studies [1]. Conversely, constructive conflict, defined as the cooperative resolution of disagreements through mutual respect and problem-solving, has been consistently linked to enhanced emotional security and improved psychological well-being in children [2]. This theoretical lens underscores the importance of examining not only the adverse impacts of destructive conflict but also the potential benefits of constructive conflict on parenting and child development outcomes.

**Figure 1:**
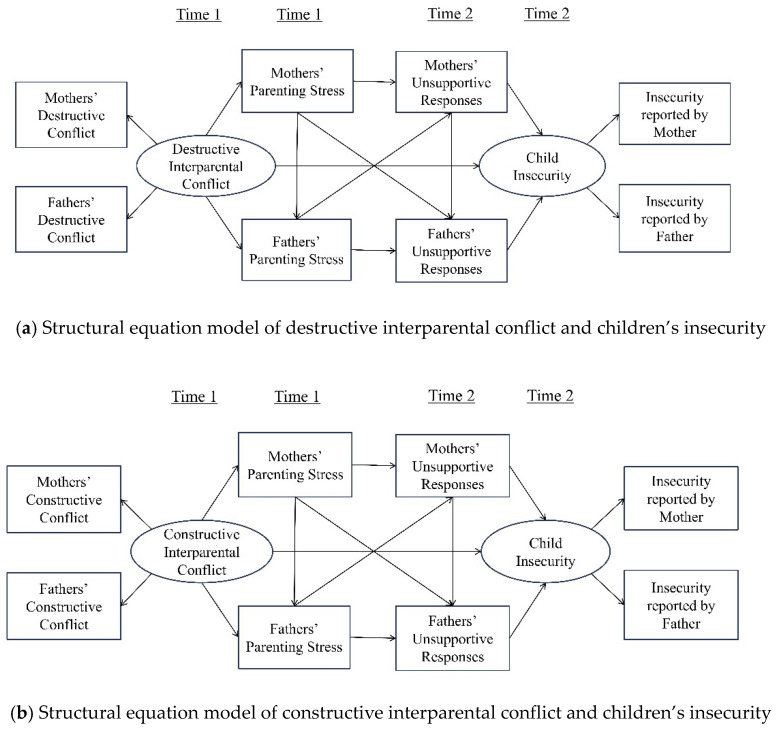
Research model.

The present study builds on the existing literature to examine the associations between constructive and destructive conflict, parenting stress, unsupportive reactions, and children’s insecurity in the context of interparental conflict. To address the complexity of these relationships, this study proposes six hypotheses of the research model (Figure 1).

First, the initial hypothesis is that there will be a positive correlation between destructive interparental conflict and maternal and paternal parenting stress, and a negative correlation between constructive interparental conflict and parenting stress. Second, it is predicted that parenting stress will be associated with higher levels of unsupportive reactions at the six-month follow-up. Third, we hypothesize that unsupportive reactions from mothers and fathers will be associated with increased insecurity in children in the context of interparental relationships. These three hypotheses primarily address the spillover effects between the mother–father and parent–child relationships. The fourth hypothesis concerns the crossover effect, which posits that an increase in parenting stress experienced by one parent will result in a corresponding increase in unsupportive parenting behaviors exhibited by the other parent.

The fifth hypothesis posits that destructive conflict will exert a direct effect on children’s insecurity six months later, whereas constructive conflict will be directly associated with lower levels of child insecurity over the same period. Finally, the sixth hypothesis postulates that destructive conflict will be indirectly associated with elevated levels of child insecurity through its impact on mothers’ and fathers’ parenting stress and unsupportive reactions. Conversely, constructive conflict will be indirectly associated with reduced levels of child insecurity through the same pathway.

The present study builds upon existing research, primarily in Western populations, to examine these processes through two dual mediation models in a sample of Korean 4.5-year-old children. By examining these dynamics in a longitudinal framework, this study aims to provide a deeper understanding of the impact of different types of interparental conflict on children’s insecurity, both directly and indirectly, through their effects on parenting stress and unsupportive responses.

## 2. Method

### 2.1. Participants and Procedure

This study explored how children’s insecurity is intertwined with interparental conflict, parenting stress, and parents’ unsupportive responses in coping with children’s negative emotions in Korean families. The participants were 210 couples of parents with children aged 3–5 years living in Korea. Two surveys were conducted with an interval of 6–7 months between them. The first survey was conducted from November 2018 to February 2019, and the second survey was conducted from June to August 2019. The first survey investigated interparental conflict and parenting stress, whereas the second investigated parents’ unsupportive responses and children’s insecurity. The couples and families who participated in the first survey were the same as those who participated in the second, with a total of 210 couples. The guardians who participated in this study were their parents. Non-responses to the questionnaire were processed using multiple imputation methods [32]. In the first step, 5 data sets were created by imputing missing values based on fully conditional specification (FCS), a widely used multiple imputation method. Next, the analysis of each of the 5 complete data sets produced parameter estimates and standard errors. Finally, these results were combined using Rubin’s rule [33,34]. The final number of participants were 159 dyads. Informed consent for research participation was obtained, and the questionnaire was completed by visiting the family in person or via the Internet. A gift certificate worth $10 was provided to the participating families. The age of their mothers, their parents, ranged from 22 to 48 years (M = 36.58, SD = 3.66), and the age of their fathers ranged from 22 to 52 years (M = 38.11, SD = 4.08). The median monthly family income of the participants was between KRW 4,070,000 (USD 3064) and KRW 5,400,000 (USD 4065). The children were 3–5 years old (M = 4.53, SD = 1.10), comprising 78 girls and 81 boys. Of the mothers, 80.5% had a bachelor’s degree or higher, but 49.1% were on leave or unemployed. Of the fathers, 83.0% had a bachelor’s degree or higher, and most (98.7%) were employed.

### 2.2. Measurement

#### 2.2.1. Interparental Conflict

In the first survey, the Marital Conflict and Problem-Solving Scale (CPS) [35], which was modified by Lee and Seo [36], was used to fit Korean culture. The destructive interparental conflict strategy subscales included avoidance/capitulation, verbal aggression, physical aggression, stonewalling, and child involvement. The constructive interparental conflict strategy subscales were measured through cooperation. It comprises 44 items. Responses to all items in the destructive and constructive interparental conflict strategy subscales were evaluated using a 4-point Likert scale (never = 0, often = 3). The mothers’ destructive and constructive interparental conflict scale was formed by averaging each item from the self-report and spouse (father) report to form a composite score, and the fathers’ destructive and constructive interparental conflict scale was also formed by averaging each item from the self-report and spouse (mother) report to form a composite score. A higher score indicated a higher level of conflict experienced by the couple. The internal reliability (Cronbach’s alpha) of the sum of the destructive interparental conflict strategy subscale was 0.939 for mothers (sum of self-reports and spouse’s perceived partner reports) and 0.925 for fathers (sum of self-reports and spouse’s perceived partner reports). The internal reliability (Cronbach’s alpha) of the constructive interparental conflict cooperation scale was 0.877 for mothers (sum of self-reports and spouse’s perceived partner reports) and 0.881 for fathers (sum of self-reports and spouse’s perceived partner reports).

#### 2.2.2. Parenting Stress

To assess parenting stress in couples, the “Parenting Stress Scale” developed in Korean by Kim and Kang [37] was used. This scale is designed to identify the stress experienced by parents in the process of raising children and consists of 11 items corresponding to the sub-factor of “Burden and distress in performing parental roles” in the original scale. Examples of items include “When I am tired and my child whines to play, I feel annoyed, “It seems difficult to be friendly and warm with the child”, and so on. Responses were recorded on a 5-point Likert scale (not at all = 1, very much = 5). The stress levels of the mothers and fathers were measured using self-reported averages. A higher score indicated a higher level of stress experienced by couples in the process of raising children. Cronbach’s α of parenting stress was 0.877 for mothers and 0.866 for fathers.

#### 2.2.3. Parental Unsupportive Responses

Parental unsupportive responses were measured using the Coping with Children’s Negative Emotions Scale (CCNES) developed by Fabes, Eisenberg, and Bernzweig [38], which was modified by Ahn [39] to fit Korean culture. The categories of situations in which children experience negative emotions and the unsupportive responses of mothers and fathers comprise 36 items in total, with subareas of distress, punishment, and minimization reactions [38]. Each item was rated on a 7-point Likert scale (not at all = 1, always = 7). In this study, the minimization reactions, which had low correlations and low factor loading criteria among the subdomains of unsupportive responses, were removed. The items of the distress and punishment reaction factors were self-reported and averaged to obtain a composite score. A higher score indicated more unsupportive responses toward the child. Cronbach’s α of unsupportive responses was 0.887 for mothers and 0.898 for fathers.

#### 2.2.4. Children’s Insecurity

To measure children’s insecurity, we used the Security in the Marital Subsystem Scale-Parent Report Inventory (SIMS-PR [40]), a scale modified by Lee and Seo [41] to fit Korean culture. It consists of 28 items in four subareas (overt emotional reactivity, behavioral dysregulation, overt avoidance, and overt involvement) that describe children’s reactions to interparental conflict. Children’s insecurity is explained by overt emotional reactivity and behavioral dysregulation [36]. Each item is rated on a 5-point Likert scale (not at all = 1, very much = 5). Children’s insecurity was calculated by averaging the items from the mothers’ and fathers’ reports to form a composite score. The higher the score, the higher the child’s insecurity was. Cronbach’s α of the children’s insecurity was 0.870 for mothers and 0.865 for fathers.

#### 2.2.5. Data Analysis

A structural equation model (SEM) was applied to verify the relationships between destructive and constructive interparental conflict, parenting stress, parents’ unsupportive responses, and children’s insecurity. First, the mean, standard deviation, skewness, and kurtosis of the main observed variables were confirmed through a basic statistical analysis, and a correlation analysis between the variables was conducted.

Second, to verify the goodness of fit of the research model, the *χ*^2^ value and GFI, CFI, IFI, RMR, and RMSEA values were comprehensively reviewed. Since the *χ*^2^ value is sensitive to the sample size and tends to easily reject data, other goodness-of-fit indices, known as good indices for the simplicity and explanatory power of the model, were utilized more [42]. GFI, CFI, and IFI are interpreted as a good fit if they are 0.9 or higher, a generally adequate fit if they are 0.8 or higher, and RMR and RMSEA are interpreted as a good fit if they are 0.05 or lower, and a generally adequate fit if they are 0.08 (or 0.10) [43,44,45].

In particular, this study used a multiple imputation method to handle missing values, assuming the missing data mechanism as randomly missing. Among the various imputation methods, fully conditional specification (FCS) was employed. Specifically, five data sets were generated through iterative imputations, analyzed to obtain parameter estimates and standard errors, and combined using Rubin’s rule [33,34,46].

Third, to confirm the mediating effect of parenting stress and parental unsupportive responses on the relationship between destructive and constructive interparental conflict and children’s insecurity, the bootstrapping procedure proposed by Shrout and Bolger [47] was followed. This method estimates the parameters using 5000 bootstrapping samples generated by random sampling from the original data, and the confidence interval is set to 95%. The significance of the mediating effect was confirmed based on whether the size of the indirect effect included zero, as set by the null hypothesis in the 95% confidence interval. Bootstrapping was conducted by setting phantom variables to verify the significance of the individual indirect effects. When using bootstrapping when there are two or more mediating variables, only the overall mediating effect and significance are presented, and the indirect effects for individual mediators are not presented; therefore, phantom variables that do not affect the fit or parameter values were set. All data were analyzed using SPSS STATISTICS 22.0 and AMOS 22.0.

## 3. Results

### 3.1. Descriptive Statistics

The mean and standard deviation of the main observed variables were obtained through basic statistical analysis, and the absolute values of skewness and kurtosis were examined to ensure the normality of all variables. Regarding the correlation analysis of the observed variables (Table 1), all the variables generally showed significant correlations. Additionally, the absolute values of all individual observed variables did not exceed two for skewness and seven for kurtosis, which were suitable for the normal distribution standard [48].

### 3.2. Model Fit Verification

#### 3.2.1. Destructive Interparental Conflict Model

**Figure 2:**
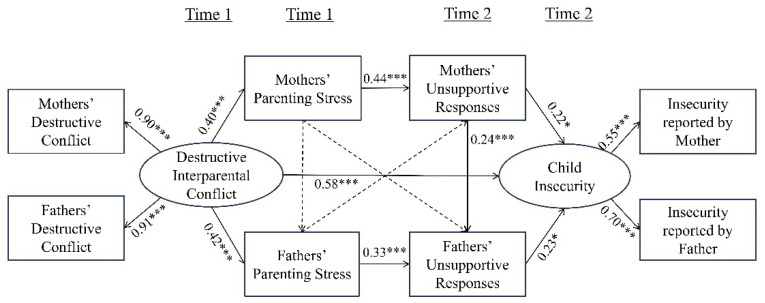
Structural equation model of destructive interparental conflict and children’s insecurity. Model fit: *χ*^2^ (16, *N* = 159) = 35.395, RMR = 0.029, GFI = 0.948, IFI = 0.952, CFI = 0.951, RMSEA = 0.088. Standardized estimates are shown as * *p* < 0.05, *** *p* < 0.001.

An SEM was applied to verify the relationships between destructive interparental conflict, parenting stress, parents’ unsupportive responses, and children’s insecurity. To select a simple final model, three insignificant paths in the research model—the effect of mothers’ stress on fathers’ stress, the effect of mothers’ stress on fathers’ unsupportive responses, and the effect of fathers’ stress on mothers’ unsupportive responses—were removed and set as the final model. The fit index of the destructive final model was *χ*^2^ (16, *N* = 159) = 35.395 ** (*p* < 0.004), RMR = 0.029, GFI = 0.948, IFI = 0.952, CFI = 0.951, and RMSEA = 0.088, which were generally good [43,44,45]. The results of checking each path estimate of the final model (Figure 2) were as follows:

**Hypothesis** **1** **(H1)** *proposed that destructive interparental conflict has a positive effect on parenting stress. Destructive interparental conflict had a positive effect on parenting stress for both mothers (β = 0.405, p < 0.001) and fathers (β = 0.423, p < 0.001).*

**Hypothesis** **2** **(H2)** *stated that parenting stress has a positive effect on unsupportive responses. Parenting stress had a positive effect on unsupportive responses in mothers (β = 0.440, p < 0.001) and fathers (β = 0.328, p < 0.001).*

**Hypothesis** **3** **(H3)** *proposed that unsupportive responses positive* *ly affect children’s insecurity. It had a positive effect on children’s insecurity among mothers (β = 0.217, p < 0.024) and fathers (β = 0.233, p < 0.016).*

**Hypothesis** **4** **(H4)** *concerns crossover effects. It was assumed that mothers’ parenting stress would have a positive effect on fathers’ parenting stress, but no significant effect was found. However, mothers’ unsupportive responses had a positive effect on fathers’ unsupportive responses (β = 0.240, p < 0.001). Mothers’ parenting stress did not significantly affect fathers’ unsupportive responses, and fathers’ parenting stress did not significantly affect mothers’ unsupportive responses.*

**Hypothesis** **5** **(H5)** *stated that destructive interparental conflict has a direct positive effect on children’s insecurity (β = 0.583, p < 0.001).*

#### 3.2.2. Constructive Interparental Conflict Model

**Figure 3:**
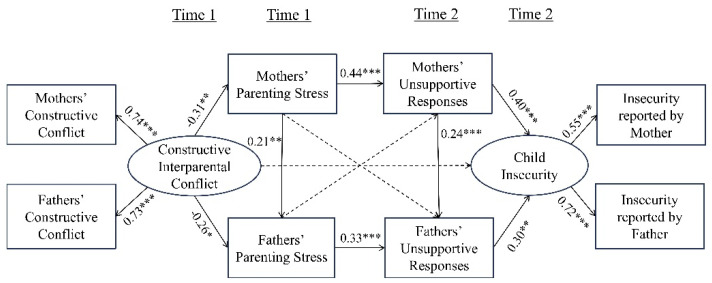
Structural equation model of constructive interparental conflict and children’s insecurity. Model fit: *χ*^2^ (16, *N* = 159) = 34.054 (*p* < 0.005), RMR = 0.031, GFI = 0.950, IFI = 0.923, CFI = 0.919, RMSEA = 0.085. Standardized estimates are presented as * *p* < 0.05, ** *p* < 0.01, *** *p* < 0.001.

An SEM was applied to verify the relationships between constructive interparental conflict, parenting stress, unsupportive responses, and child insecurity. To select a simple final model, three insignificant paths in the research model–the effect of mothers’ stress on fathers’ unsupportive responses, the effect of fathers’ stress on mothers’ unsupportive responses, and the effect path from constructive interparental conflict on children’s insecurity–were removed and set as the final model. The fit index of the constructive final model was *χ*^2^ (16, *N* = 159) = 34.054 ** (*p* < 0.005), RMR = 0.031, GFI = 0.950, IFI = 0.923, CFI = 0.919, and RMSEA = 0.085, which was generally good [43,44,45]. The results of checking each path’s estimate of the final model (Figure 3) were as follows:

**Hypothesis** **1** **(H1)** *proposed that constructive interparental conflict negative* *ly affects parenting stress. Constructive interparental conflict had a negative effect on parenting stress for mothers (β = −0.305, p < 0.003) and fathers (β = −0.255, p < 0.013).*

**Hypothesis** **2** **(H2)** *stated that parenting stress has a positive effect on unsupportive responses. It had a positive effect on unsupportive responses for mothers (β = 0.440, p < 0.001) and fathers (β = 0.326, p < 0.001).*

**Hypothesis** **3** **(H3)** *proposed that unsupportive responses positive* *ly affect children’s insecurity. It had a positive effect on children’s insecurity for mothers (β = 0.400, p < 0.001) and fathers (β = 0.296, p < 0.007).*

**Hypothesis** **4** **(H4)** *concerns crossover effect* *s. Mothers’ parenting stress had a positive effect on fathers’ parenting stress (β = 0.212, p < 0.009). In addition, mothers’ unsupportive responses had a positive effect on fathers’ unsupportive responses (β = 0.239, p < 0.001). Mothers’ stress did not have a significant effect on fathers’ unsupportive responses, and fathers’ stress did not have a significant effect on mothers’ unsupportive responses.*

### 3.3. Mediation Model Verification

#### 3.3.1. Destructive Interparental Conflict Mediation Model

The results of Hypothesis 6 are confirmed as follows. As shown in Table 2, in order to obtain the individual indirect mediating effects of parenting stress and unsupportive responses on the relationship between destructive interparental conflict and children’s insecurity, a bootstrapping procedure was conducted by setting up phantom variables.

As shown in Figure 2 and Table 3, the sequential mediating effects of parenting stress and unsupportive responses in all mediation relationships between destructive interparental conflict and children’s insecurity were significant because the 95% confidence intervals did not include 0.

The sequential mediating effect of mothers’ parenting stress and unsupportive responses on the relationship between destructive interparental conflict and children’s insecurity was also significant (*B* = 0.036, *p* < 0.04). This shows that destructive interparental conflict can increase mothers’ parenting stress, mothers’ parenting stress increases mothers’ unsupportive responses, and mothers’ unsupportive responses increase children’s insecurity.

The sequential mediation effect of fathers’ parenting stress and unsupportive responses on the relationship between destructive interparental conflict and children’s insecurity was also significant (*B* = 0.030, *p* < 0.043). This shows that destructive interparental conflict can increase fathers’ parenting stress, fathers’ parenting stress increases fathers’ unsupportive responses, and fathers’ unsupportive responses increase the level of children’s insecurity.

The sequential mediating effects of mothers’ parenting stress, mothers’ unsupportive responses, and fathers’ unsupportive responses on the relationship between destructive interparental conflict and children’s insecurity were also significant (*B* = 0.009, *p* < 0.032). Destructive interparental conflict can increase mothers’ parenting stress, mothers’ parenting stress can increase mothers’ unsupportive responses, mothers’ unsupportive responses can increase fathers’ unsupportive responses, and fathers’ unsupportive responses can increase children’s insecurity. In conclusion, destructive interparental conflict can increase insecurity among children.

#### 3.3.2. Constructive Interparental Conflict Mediation Model

The results of Hypothesis 6 are confirmed as follows. As shown in Table 4, in order to obtain the individual indirect mediating effects of parenting stress and unsupportive responses on the relationship between constructive interparental conflict and children’s insecurity, a bootstrapping procedure was conducted by setting up phantom variables.

As shown in Figure 3 and Table 5, the sequential mediating effects of parenting stress and unsupportive responses in all mediation relationships between constructive interparental conflict and children’s insecurity were significant because the 95% confidence intervals did not include 0.

The sequential mediation effects of mothers’ parenting stress and mothers’ unsupportive responses on the relationship between constructive interparental conflict and children’s insecurity were also significant (*B* = −0.054, *p* < 0.005). This shows that constructive interparental conflict can lower mothers’ parenting stress, mothers’ parenting stress increases mothers’ unsupportive responses, and mothers’ unsupportive responses increase the level of children’s insecurity.

The sequential mediation effect of fathers’ parenting stress and fathers’ unsupportive responses on the relationship between constructive interparental conflict and children’s insecurity was also significant (*B* = −0.025, *p* < 0.017). This shows that constructive interparental conflict can lower fathers’ parenting stress, fathers’ parenting stress increases fathers’ unsupportive responses, and fathers’ unsupportive responses increase the level of children’s insecurity.

The sequential mediation effects of mothers’ parenting stress, mothers’ unsupportive responses, and fathers’ unsupportive responses in the relationship between constructive interparental conflict and children’s insecurity were also significant (*B* = −0.009, *p* < 0.015). Constructive interparental conflict can lower mothers’ parenting stress, mothers’ parenting stress can increase mothers’ unsupportive responses, mothers’ unsupportive responses can increase fathers’ unsupportive responses, and fathers’ unsupportive responses can increase children’s insecurity.

The sequential mediation effects of mothers’ parenting stress, fathers’ parenting stress, and fathers’ unsupportive responses in the relationship between constructive interparental conflict and children’s insecurity were also significant (*B* = −0.006, *p* < 0.015). Constructive interparental conflict can lower mothers’ parenting stress, mothers’ parenting stress can increase fathers’ parenting stress, fathers’ parenting stress can increase fathers’ unsupportive responses, and fathers’ unsupportive responses can increase children’s insecurity. In conclusion, constructive interparental conflict can reduce children’s insecurity.

## 4. Discussion

These findings underscore the pivotal role of interparental conflict, whether destructive or constructive, in shaping parenting stress and the subsequent unsupportive reactions parents exhibit toward their children. The first hypothesis, which proposed a positive correlation between destructive interparental conflict and maternal and paternal parenting stress and a negative correlation between constructive interparental conflict and parenting stress, was fully supported. These findings are consistent with previous studies, indicating that the quality of interparental conflict has a direct impact on stress levels [3].

Additionally, support was found for the second hypothesis. Higher parenting stress was associated with increased unsupportive responses from both mothers and fathers at the six-month follow-up, consistent with the spillover effect of stress in the parent–child relationship, which has been demonstrated to result in less effective and negative parenting behaviors [49]. Support for the third hypothesis indicated that unsupportive responses from both parents were associated with elevated insecurity levels in children in interparental relationships. These findings illustrate the necessity of examining both the interparental relationship and spillover effects in parent–child relationships. Previous studies that tested Abidin’s stress model predominantly employed cross-sectional research designs that were unable to ascertain the effects of parenting stress over time [50]. It can be reasonably assumed that parenting stress affects children’s behavior. However, it is also evident that children’s behaviors affect their parents. Moreover, meta-studies have demonstrated that in the majority of empirical studies, changes in parenting and child behavior occur concurrently within families. However, these changes were not correlated over a period exceeding six months [51]. Nevertheless, the findings of the present study elucidate the direction of the effects postulated by Abidin’s model. They demonstrated that parenting stress affects unsupportive parenting behaviors six months later and the consequences of these parenting behaviors on children’s insecurity.

The fourth hypothesis, which addresses crossover effects, was only partially supported. In both the destructive and constructive conflict models, mothers’ unsupportive responses influenced fathers’ unsupportive parenting responses. However, in the destructive conflict model, mothers’ parenting stress did not influence fathers’ parenting stress. Moreover, there was no crossover effect of maternal and paternal parenting stress on partners’ unsupportive parenting behaviors at six months. The absence of an effect of maternal parenting stress on paternal parenting stress in the destructive conflict model may be attributed to the more pronounced direct impact of destructive conflict on paternal parenting stress than the indirect influence of maternal parenting stress. The absence of a crossover effect between parenting stress and unsupportive parenting behaviors six months later may be attributed to the short-term longitudinal study design. Prior empirical studies have indicated that the relationship between parenting stress and parenting behaviors can exhibit inconsistent crossover effects contingent on whether the study is longitudinal or cross-sectional [50,52]. These findings indicate that the cross effects between parenting stress and behaviors may become relatively weak over time.

Our fifth hypothesis was partially supported. The findings revealed that destructive conflict had a direct effect on children’s anxiety six months later. However, constructive conflict was not directly associated with lower anxiety levels in the children over the same period. The final hypothesis regarding the indirect effects was fully supported. All indirect paths proposed by the destructive and constructive conflict models were statistically significant. Partial support for the fifth hypothesis may be because of differences in the impact of destructive and constructive conflicts. If the negative effects of destructive conflict spill over to children through parenting stress and behaviors, constructive conflict may at least reduce negative parental reactions; however, the effects may be weaker than those of destructive conflict. The weaker spillover effects of constructive conflict compared to destructive conflict may be attributed to Korean characteristics of emotion regulation and expression. McDonald [53] posited that Koreans tend to regulate and express negative emotions in accordance with collectivistic social norms. In destructive conflict situations, they may lose their capacity for emotional control. Conversely, in constructive conflict situations, they may limit their cooperative emotional expressions to maintain this control.

In summary, this study offers significant insights into the influence of diverse forms of interparental conflict on parenting stress, unsupportive behaviors, and child insecurity within families. By examining these dynamics in a Korean sample and considering both spillover and crossover effects, this study provides a more comprehensive understanding of the pathways through which interparental conflict affects children’s development. Further research should continue to examine these relationships in different cultural contexts and explore the long-term implications of these findings for family interventions aimed at reducing child insecurity and promoting positive parenting [54].

In addition to contributing to the existing literature on interparental conflict, parenting, and child insecurity, this study has several methodological strengths. First, the study’s approach to coding both destructive and constructive behaviors during conflict represents a significant advancement in the field, as it permits a more comprehensive understanding of the impact of different conflict types on parenting stress and child outcomes. Second, the study employed reports from both mothers and fathers for all variables rather than relying on a single rapporteur. This approach reduced the potential for methodological bias and provided a more balanced and accurate representation of parenting behaviors. Third, this study addresses an important gap in the existing literature by focusing on Asian families, particularly Korean families, which have been underrepresented in research on interparental conflict and child development. As cultural perceptions of emotion regulation and expression remain disparate between Western and Eastern societies, the findings of this study are particularly meaningful and provide valuable insights into the distinctive dynamics of Korean family life.

Although this study has notable methodological strengths, it is not without limitations. First, all variables were measured via self-reporting, which may have introduced a degree of bias. Future research should consider direct observations of the Korean population to gain a more nuanced understanding of the interactions in question. Second, although this study focused solely on negative parenting styles to investigate the consequences of interparental conflict, future research could be enhanced by examining whether constructive conflict encourages positive parenting styles, such as warmth and sensitivity, which are acknowledged as protective factors against child insecurity. Finally, the sample used in this study was relatively homogeneous and comprised only Korean participants, which may restrict the generalizability of our findings. It would be beneficial for future studies to aim to include a larger and more diverse sample in order to increase the applicability of the findings.

The findings of this study have significant implications for advancing our understanding of the intricate relationship between interparental conflict, parenting stress, parenting behaviors, and children’s insecurity. By adopting a dual mediation model, this study offers a unique longitudinal perspective on dyadic interactions between mothers and fathers, elucidating the ways in which these relationships evolve and influence child development. These findings underscore the need to view the family as a system in which constructive and destructive conflicts have disparate effects on family functioning.

In conclusion, the findings support the notion that it is the manner in which conflict is managed rather than merely the existence of conflict that is crucial for child development [21]. The insights gained from this study into the impact of conflict on parenting practices highlight the importance of considering both spillover and crossover effects when attempting to comprehend the pathways through which interparental conflict affects children’s insecurity. As this study illustrates, examining these dynamics within the distinctive cultural context of Korean families offers valuable insights into the field while encouraging further research into the cultural dimensions of parenting and child development.

## Figures and Tables

**Table 1:** Descriptive statistics and correlations between the study variables.

Variable	1	2	3	4	5	6	7	8	9	10
1. M_DC	1									
2. F_DC	0.817 **	1								
3. M_CC	−0.462 **	−0.344 **	1							
4. F_CC	−0.323 **	−0.431 **	0.540 **	1						
5. M_PS	0.373 **	0.342 **	−0.245 **	−0.202 *	1					
6. F_PS	0.357 **	0.383 **	−0.219 **	−0.252 **	0.289 **	1				
7. M_UR	0.368 **	0.371 **	−0.206 **	−0.148	0.440 **	0.238 **	1			
8. F_UR	0.176 *	0.245 **	−0.168 *	−0.200 *	0.085	0.380 **	0.314 **	1		
9. M_CI	0.372 **	0.366 **	−0.073	−0.086	0.315 **	0.171 *	0.339 **	0.142	1	
10. F_CI	0.410 **	0.458 **	−0.163 *	−0.160 *	0.227 **	0.385 **	0.320 **	0.353 **	0.399 **	1
M	0.947	0.967	2.579	2.541	2.530	2.201	2.889	2.721	1.896	1.950
SD	0.348	0.345	0.399	0.422	0.713	0.671	0.729	0.824	0.537	0.606
*S*	0.774	0.187	−1.388	−1.102	0.491	0.262	0.229	−0.206	0.425	−0.130
*K*	1.321	−0.030	2.496	1.136	0.007	−0.255	−0.418	0.966	−0.296	1.433

Note. *N* = 159. M_DC, mothers’ destructive conflicts; F_DC, fathers’ destructive conflict; M_CC, mothers’ constructive conflict; F_CC, fathers’ constructive conflict; M_PS, mothers’ parenting stress; F_PS, fathers’ parenting stress; M_UR, mothers’ unsupportive responses; F_UR, fathers’ unsupportive responses; M_CI, child insecurity reported by mother; F_CI, child insecurity reported by father. * *p* < 0.05, ** *p* < 0.01.

**Table 2:** Direct effect, indirect effect, and total effect of destructive interparental conflict.

Pathway			Direct Effect	Indirect Effect	Total Effect
DC	→	M_PS	0.927 ***		0.927 ***
DC	→	F_PS	0.912 ***		0.912 ***
DC	→	M_UR		0.417 ***	0.417 ***
DC	→	F_UR		0.474 ***	0.474 ***
DC	→	CI	0.545 ***	0.076 ***	0.621 ***
M_PS	→	M_UR	0.450 ***		0.450 ***
M_PS	→	F_UR		0.121 **	0.121 **
M_PS	→	CI		0.049 **	0.049 **
F_PS	→	F_UR	0.397 ***		0.397 ***
F_PS	→	CI		0.033 ^†^	0.033 ^†^
M_UR	→	F_UR	0.268 **		0.268 **
M_UR	→	CI	0.086 ^†^	0.022 *	0.109 **
F_UR	→	CI	0.083 ^†^		0.083 ^†^

Note. DC, destructive interparental conflict; M, mother; F, father; CI, child insecurity; UR, unsupportive response. ^†^ *p* < 0.1, * *p* < 0.05, ** *p* < 0.01, *** *p* < 0.001. *N* = 159, and the number of bootstrapping samples was 5000. All the figures above are unstandardized coefficients.

**Table 3:** Results of path verification of multiple mediation effects of destructive interparental conflict.

Pathway									Indirect Effect	95% CI LLCI, ULCI
DC	→	M_PS	→	M_UR	→	CI			0.036	[0.001, 0.111]
DC	→	F_PS	→	F_UR	→	CI			0.030	[0.001, 0.070]
DC	→	M_PS	→	M_UR	→	F_UR	→	CI	0.009	[0.001, 0.031]

Note. DC, destructive interparental conflict; M, mother; F, father; CI, child insecurity; UR, unsupportive responses; LLCI, lower-limit confidence interval; ULCI, upper-limit confidence interval.

**Table 4:** Direct effect, indirect effect, and total effect of constructive interparental conflict.

Pathway			Direct Effect	Indirect Effect	Total Effect
CC	→	M_PS	−0.735 **		−0.735 **
CC	→	F_PS	−0.579 **	−0.146 **	−0.726 ***
CC	→	M_UR		−0.331 **	−0.331 **
CC	→	F_UR		−0.377 ***	−0.377 ***
CC	→	CI		−0.094 ***	−0.094 ***
M_PS	→	F_PS	0.199 *		0.199 *
M_PS	→	M_UR	0.450 ***		0.450 ***
M_PS	→	F_UR		0.200 ***	0.200 ***
M_PS	→	CI		0.094 ***	0.094 ***
F_PS	→	F_UR	0.397 ***		0.397 ***
F_PS	→	CI		0.042 *	0.042 *
M_UR	→	F_UR	0.268 **		0.268 **
M_UR	→	CI	0.162 **	0.029 *	0.191 ***
F_UR	→	CI	0.107 *		0.107 *

Note. CC, constructive interparental conflict; M, mother; F, father; CI, child insecurity; UR, unsupportive response. * *p* < 0.05, ** *p* < 0.01, *** *p* < 0.001. *N* = 159, and the number of bootstrapping samples was 5000. All the figures above are unstandardized coefficients.

**Table 5:** Results of path verification of multiple mediation effects of constructive interparental conflict.

Pathway									Indirect Effect	95% CI LLCI, ULCI
CC	→	M_PS	→	M_UR	→	CI			−0.054	[−0.171, −0.006]
CC	→	F_PS	→	F_UR	→	CI			−0.025	[−0.089, −0.003]
CC	→	M_PS	→	M_UR	→	F_UR	→	CI	−0.009	[−0.037, −0.001]
CC	→	M_PS	→	F_PS	→	F_UR	→	CI	−0.006	[−0.024, −0.001]

Note. CC = constructive interparental conflict; M = mother; F = father; CI = child insecurity; UR = unsupportive responses; LLCI = lower-limit confidence interval; ULCI = upper-limit confidence interval.

## Data Availability

The data presented in this study are available upon request from the corresponding author.
